# 3,3′-Dibromo-6,6′-dimethoxy­biphenyl-2,2′-dicarboxylic acid ethanol monosolvate

**DOI:** 10.1107/S1600536810013279

**Published:** 2010-04-24

**Authors:** Xiao Zhang, Long Li, Le Zhou, Baoming Ji

**Affiliations:** a Northwest Agriculture and Forest University, Yangling 712100, People’s Republic of China; b College of Chemistry and Chemical Engineering, Luoyang Normal University, Luoyang 471022, People’s Republic of China

## Abstract

In the title compound, C_16_H_12_Br_2_O_6_·C_2_H_5_OH, the two benzene rings are twisted by 80.64 (5)° and the carboxyl groups form dihedral angles of 72.48 (3) and 89.41 (2)° with the corresponding benzene rings. In the crystal structure, the biphenyl mol­ecules are connected by inter­molecular O—H⋯O and O—H⋯Br hydrogen bonds, resulting in a chain along the *b* axis.

## Related literature

For complexes containing diphenic acids, see: Wang *et al.* (2007 ▶); Yang *et al.* (2007 ▶). For the synthesis of the title compound, see: Choi *et al.* (2007 ▶).
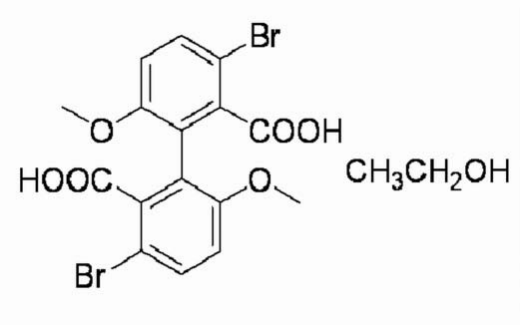


## Experimental

### 

#### Crystal data

C_16_H_12_Br_2_O_6_·C_2_H_6_O
*M* *_r_* = 506.14
Monoclinic, 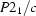

*a* = 9.9872 (9) Å
*b* = 23.230 (2) Å
*c* = 8.3967 (7) Å
β = 90.143 (1)°
*V* = 1948.1 (3) Å^3^
*Z* = 4
Mo *K*α radiation
μ = 4.20 mm^−1^
*T* = 296 K
0.41 × 0.34 × 0.32 mm

#### Data collection

Bruker APEXII CCD diffractometer
Absorption correction: multi-scan (*SADABS*; Sheldrick, 1996 ▶) *T*_min_ = 0.278, *T*_max_ = 0.347
14693 measured reflections
3606 independent reflections
2825 reflections with *I* > 2σ(*I*)
*R*_int_ = 0.030

#### Refinement

*R*[*F* ^2^ > 2σ(*F* ^2^)] = 0.029
*wR*(*F* ^2^) = 0.069
*S* = 1.02
3606 reflections
250 parameters
H-atom parameters constrained
Δρ_max_ = 0.35 e Å^−3^
Δρ_min_ = −0.52 e Å^−3^

### 

Data collection: *APEX2* (Bruker, 2004 ▶); cell refinement: *SAINT* (Bruker, 2004 ▶); data reduction: *SAINT*; program(s) used to solve structure: *SHELXS97* (Sheldrick, 2008 ▶); program(s) used to refine structure: *SHELXL97* (Sheldrick, 2008 ▶); molecular graphics: *SHELXTL* (Sheldrick, 2008 ▶) and *DIAMOND* (Brandenburg, 2006 ▶); software used to prepare material for publication: *SHELXTL* and *PLATON* (Spek, 2009 ▶).

## Supplementary Material

Crystal structure: contains datablocks global, I. DOI: 10.1107/S1600536810013279/hg2670sup1.cif

Structure factors: contains datablocks I. DOI: 10.1107/S1600536810013279/hg2670Isup2.hkl

Additional supplementary materials:  crystallographic information; 3D view; checkCIF report

## Figures and Tables

**Table 1:** Hydrogen-bond geometry (Å, °)

*D*—H⋯*A*	*D*—H	H⋯*A*	*D*⋯*A*	*D*—H⋯*A*
O4—H4*A*⋯O7	0.82	1.79	2.594 (3)	165
O6—H6⋯O3^i^	0.82	1.89	2.711 (3)	174
O7—H7⋯O5^ii^	0.82	2.07	2.879 (3)	167
O7—H7⋯Br1^ii^	0.82	3.04	3.445 (2)	113

Symmetry codes: (i) 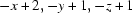; (ii) 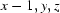.

**Fig. 1.**
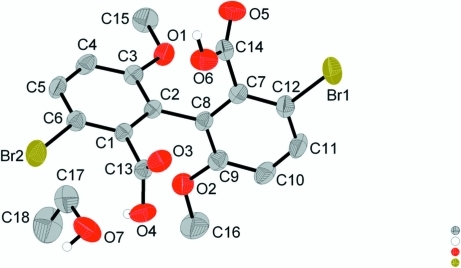
View of the title molecule with the atom numbering scheme and 30% probability displacement ellipsoids for non-hydrogen atoms.

**Fig. 2.**
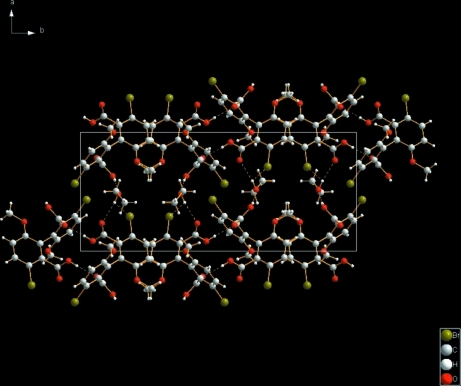
View of a 2D supramolecular network with 1D channels along the c axis. (O—H···O and O—H···Br hydrogen bonds are indicated as broken lines).

## supplementary crystallographic information

### Comment

Diphenic acid and its derivatives have been proved to be a kind of multifunctional and flexible ligand in the construction of complexes possessing novel and interesting topological structures (Choi *et al.* 2007). Our interest in these compounds has led us to prepare the title compound according to the literature methods (Choi *et al.* 2007). In this contribution, we report the synthesis and crystal structure of the title compound.

The molecule of the title compound (Fig. 1.), is built up form one benzene ring connected to the other benzene ring through the 2 and 2' carbon atoms, in which the bond lengths and angles are within ranges as reported by Wang *et al.*. (2007). In the crystal structure, except the carboxyl group, both methoxyl group and bromino group lie in the corresponding benzene ring plane, with an r.m.s. deviation of 0.0180 (1) Å and 0.0124 (1) Å.respectively. And, the dihedral angle between the two benzene rings is 80.64 (5)°. It must be pointed out that the striking feature of the title compound is the interesting arrangement of the tilte molecules, which connected each other by the formation of intermolecular O—H···O hydrogen bonds to form one-dimensional chain along the *b* axis (Fig.2.). Interestingly, the solvent molecules interact with the carboxylate oxygen atoms and bromine atoms from the chains via O—H···O and O—H···Br hydrogen bonds respectively, resulting in the formation of a 2D supramolecular network with 1D channels along the *c* axis. Detail hydrogen bonds are given in Table 1.

### Experimental

2,2'-Dimethoxy-6,6'-Diacetylbiphenyl (0.298 mg, 1 mmol) was suspended and stirred in 1,4-dioxane (24 ml). NaOH (1.76 g, 30.1 mmol) was dissolved in 11.8 ml of water. At 0 °C, bromine (0.80 ml) was added to the NaOH solution which was stirred for 15 min. The NaOBr solution was added gradually to the 1,4-dioxane solution at room temperature, then was stirred at 60 °C for 2 h and cooled to room temperature. The mixture was acidified with *conc*. HCl (pH < 2) and filtered, washed with water (5 × 100 ml). The products were dried under vacuum, gave the title compounds as white solid (0.253 g, yield 84%).

### Refinement

All H atoms were placed in calculated positions (C—H 0.93 ~0.97 Å, O—H 0.82 Å) and were included in the refinement in the riding model approximation, with *U*ĩso~(H) set to 1.2 ~1.5Ueq (C, O)

### Crystal data

**Table:**

C_16_H_12_Br_2_O_6_·C_2_H_6_O	*F*(000) = 1008
*M**_r_* = 506.14	*D*_x_ = 1.726 Mg m^−^^3^
Monoclinic, *P*2_1_/*c*	Mo *K*α radiation, λ = 0.71073 Å
Hall symbol: -P 2ybc	Cell parameters from 4208 reflections
*a* = 9.9872 (9) Å	θ = 2.6–24.1°
*b* = 23.230 (2) Å	µ = 4.20 mm^−^^1^
*c* = 8.3967 (7) Å	*T* = 296 K
β = 90.143 (1)°	Block, colourless
*V* = 1948.1 (3) Å^3^	0.41 × 0.34 × 0.32 mm
*Z* = 4	

### Data collection

**Table:**

Bruker APEXII CCD diffractometer	3606 independent reflections
Radiation source: fine-focus sealed tube	2825 reflections with *I* > 2σ(*I*)
graphite	*R*_int_ = 0.030
phi and ω scans	θ_max_ = 25.5°, θ_min_ = 2.6°
Absorption correction: multi-scan (*SADABS*; Sheldrick, 1996)	*h* = −12→12
*T*_min_ = 0.278, *T*_max_ = 0.347	*k* = −28→27
14693 measured reflections	*l* = −10→10

### Refinement

**Table:**

Refinement on *F*^2^	Primary atom site location: structure-invariant direct methods
Least-squares matrix: full	Secondary atom site location: difference Fourier map
*R*[*F*^2^ > 2σ(*F*^2^)] = 0.029	Hydrogen site location: inferred from neighbouring sites
*wR*(*F*^2^) = 0.069	H-atom parameters constrained
*S* = 1.02	*w* = 1/[σ^2^(*F*_o_^2^) + (0.0296*P*)^2^ + 1.1811*P*] where *P* = (*F*_o_^2^ + 2*F*_c_^2^)/3
3606 reflections	(Δ/σ)_max_ = 0.001
250 parameters	Δρ_max_ = 0.35 e Å^−^^3^
0 restraints	Δρ_min_ = −0.51 e Å^−^^3^

### Special details

**Table:**

Geometry. All esds (except the esd in the dihedral angle between two l.s. planes)are estimated using the full covariance matrix. The cell esds are takeninto account individually in the estimation of esds in distances, anglesand torsion angles; correlations between esds in cell parameters are onlyused when they are defined by crystal symmetry. An approximate (isotropic)treatment of cell esds is used for estimating esds involving l.s. planes.
Refinement. Refinement of *F*^2^ against ALL reflections. The weighted *R*-factor wR andgoodness of fit *S* are based on *F*^2^, conventional *R*-factors *R* are basedon *F*, with *F* set to zero for negative *F*^2^. The threshold expression of*F*^2^ > σ(*F*^2^) is used only for calculating *R*-factors(gt) etc. and isnot relevant to the choice of reflections for refinement. *R*-factors basedon *F*^2^ are statistically about twice as large as those based on *F*, and *R*-factors based on ALL data will be even larger.

### Fractional atomic coordinates and isotropic or equivalent isotropic displacement parameters (Å^2^)

**Table:**

	*x*	*y*	*z*	*U*_iso_*/*U*_eq_	
Br1	1.29076 (3)	0.321685 (15)	0.54701 (4)	0.05407 (12)	
Br2	0.56847 (4)	0.521511 (14)	0.30185 (4)	0.05610 (12)	
C1	0.7693 (3)	0.43425 (10)	0.2858 (3)	0.0278 (6)	
C2	0.8694 (3)	0.40203 (10)	0.2113 (3)	0.0274 (6)	
C3	0.9035 (3)	0.41516 (11)	0.0534 (3)	0.0305 (6)	
C4	0.8397 (3)	0.45929 (12)	−0.0261 (3)	0.0370 (7)	
H4	0.8629	0.4676	−0.1307	0.044*	
C5	0.7418 (3)	0.49106 (12)	0.0482 (3)	0.0385 (7)	
H5	0.6992	0.5208	−0.0061	0.046*	
C6	0.7069 (3)	0.47879 (11)	0.2029 (3)	0.0344 (6)	
C7	1.0635 (3)	0.35965 (11)	0.3670 (3)	0.0308 (6)	
C8	0.9375 (3)	0.35320 (11)	0.2973 (3)	0.0293 (6)	
C9	0.8739 (3)	0.29955 (11)	0.3056 (3)	0.0354 (6)	
C10	0.9326 (3)	0.25405 (12)	0.3876 (4)	0.0448 (7)	
H10	0.8887	0.2189	0.3952	0.054*	
C11	1.0562 (3)	0.26145 (12)	0.4574 (4)	0.0460 (8)	
H11	1.0956	0.2312	0.5128	0.055*	
C12	1.1220 (3)	0.31319 (12)	0.4460 (3)	0.0360 (6)	
C13	0.7292 (3)	0.42070 (11)	0.4544 (3)	0.0327 (6)	
C14	1.1369 (3)	0.41600 (11)	0.3540 (3)	0.0330 (6)	
C15	1.0350 (3)	0.39177 (14)	−0.1754 (3)	0.0502 (8)	
H15A	0.9570	0.3871	−0.2412	0.075*	
H15B	1.1028	0.3650	−0.2082	0.075*	
H15C	1.0682	0.4304	−0.1859	0.075*	
C16	0.6822 (4)	0.24274 (15)	0.2386 (5)	0.0773 (12)	
H16A	0.7326	0.2127	0.1882	0.116*	
H16B	0.5972	0.2468	0.1862	0.116*	
H16C	0.6682	0.2331	0.3485	0.116*	
C17	0.5058 (3)	0.37183 (15)	0.0619 (4)	0.0576 (9)	
H17A	0.5957	0.3592	0.0357	0.069*	
H17B	0.4998	0.4126	0.0382	0.069*	
C18	0.4087 (4)	0.34029 (17)	−0.0378 (5)	0.0830 (13)	
H18A	0.4097	0.3003	−0.0093	0.124*	
H18B	0.4325	0.3444	−0.1479	0.124*	
H18C	0.3206	0.3557	−0.0211	0.124*	
O1	1.0004 (2)	0.38108 (8)	−0.0119 (2)	0.0405 (5)	
O2	0.7542 (2)	0.29550 (8)	0.2283 (2)	0.0470 (5)	
O3	0.7835 (2)	0.44226 (8)	0.5689 (2)	0.0435 (5)	
O4	0.6324 (2)	0.38329 (9)	0.4742 (2)	0.0465 (5)	
H4A	0.5983	0.3758	0.3877	0.070*	
O5	1.2337 (2)	0.42307 (9)	0.2715 (3)	0.0525 (6)	
O6	1.0841 (2)	0.45596 (8)	0.4443 (3)	0.0506 (6)	
H6	1.1260	0.4860	0.4336	0.076*	
O7	0.4832 (2)	0.36322 (12)	0.2271 (3)	0.0622 (6)	
H7	0.4102	0.3767	0.2514	0.093*	

### Atomic displacement parameters (Å^2^)

**Table:**

	*U*^11^	*U*^22^	*U*^33^	*U*^12^	*U*^13^	*U*^23^
Br1	0.04607 (19)	0.0635 (2)	0.0526 (2)	0.01334 (16)	−0.01252 (15)	0.00325 (16)
Br2	0.0602 (2)	0.0550 (2)	0.0532 (2)	0.02797 (17)	0.01663 (16)	0.00940 (16)
C1	0.0313 (14)	0.0268 (13)	0.0251 (13)	−0.0016 (11)	−0.0020 (11)	0.0003 (11)
C2	0.0319 (14)	0.0245 (13)	0.0258 (13)	−0.0013 (11)	−0.0011 (11)	0.0006 (10)
C3	0.0353 (15)	0.0269 (14)	0.0292 (14)	0.0011 (11)	0.0018 (11)	−0.0029 (11)
C4	0.0467 (17)	0.0395 (16)	0.0248 (14)	0.0004 (13)	0.0050 (12)	0.0071 (12)
C5	0.0448 (17)	0.0353 (16)	0.0353 (16)	0.0063 (13)	−0.0009 (13)	0.0113 (12)
C6	0.0364 (15)	0.0330 (14)	0.0339 (15)	0.0040 (12)	0.0024 (12)	0.0006 (12)
C7	0.0381 (15)	0.0279 (14)	0.0265 (13)	0.0058 (12)	0.0064 (11)	−0.0004 (11)
C8	0.0351 (15)	0.0276 (14)	0.0251 (13)	0.0049 (11)	0.0045 (11)	0.0019 (11)
C9	0.0402 (16)	0.0306 (15)	0.0354 (15)	0.0016 (12)	0.0022 (12)	0.0006 (12)
C10	0.0538 (19)	0.0277 (15)	0.0529 (19)	−0.0013 (14)	0.0013 (15)	0.0080 (13)
C11	0.054 (2)	0.0341 (16)	0.0502 (19)	0.0091 (14)	−0.0031 (15)	0.0140 (14)
C12	0.0378 (16)	0.0400 (16)	0.0300 (14)	0.0096 (13)	0.0005 (12)	0.0025 (12)
C13	0.0358 (16)	0.0307 (14)	0.0316 (15)	0.0058 (12)	0.0043 (12)	0.0029 (12)
C14	0.0305 (15)	0.0353 (15)	0.0332 (15)	0.0045 (12)	−0.0038 (12)	0.0000 (12)
C15	0.058 (2)	0.059 (2)	0.0339 (16)	0.0082 (16)	0.0147 (14)	−0.0030 (14)
C16	0.072 (3)	0.055 (2)	0.105 (3)	−0.029 (2)	−0.025 (2)	0.018 (2)
C17	0.057 (2)	0.059 (2)	0.057 (2)	0.0043 (18)	−0.0069 (17)	0.0004 (17)
C18	0.098 (3)	0.069 (3)	0.081 (3)	−0.006 (2)	−0.036 (3)	−0.012 (2)
O1	0.0503 (12)	0.0419 (11)	0.0293 (10)	0.0118 (9)	0.0104 (9)	0.0007 (8)
O2	0.0429 (12)	0.0363 (11)	0.0619 (13)	−0.0087 (9)	−0.0098 (10)	0.0050 (10)
O3	0.0576 (13)	0.0463 (12)	0.0267 (10)	−0.0083 (10)	−0.0016 (9)	−0.0019 (9)
O4	0.0519 (13)	0.0543 (13)	0.0333 (11)	−0.0164 (11)	0.0048 (9)	0.0042 (10)
O5	0.0485 (13)	0.0472 (13)	0.0618 (14)	−0.0043 (10)	0.0195 (11)	−0.0020 (11)
O6	0.0546 (14)	0.0331 (11)	0.0641 (14)	−0.0054 (10)	0.0152 (11)	−0.0127 (10)
O7	0.0397 (13)	0.0960 (19)	0.0508 (14)	0.0005 (13)	0.0022 (10)	−0.0063 (13)

### Geometric parameters (Å, °)

**Table:**

Br1—C12	1.896 (3)	C13—O3	1.212 (3)
Br2—C6	1.895 (3)	C13—O4	1.310 (3)
C1—C6	1.393 (3)	C14—O5	1.202 (3)
C1—C2	1.398 (4)	C14—O6	1.311 (3)
C1—C13	1.506 (4)	C15—O1	1.438 (3)
C2—C3	1.403 (3)	C15—H15A	0.9600
C2—C8	1.506 (3)	C15—H15B	0.9600
C3—O1	1.366 (3)	C15—H15C	0.9600
C3—C4	1.379 (4)	C16—O2	1.424 (4)
C4—C5	1.376 (4)	C16—H16A	0.9600
C4—H4	0.9300	C16—H16B	0.9600
C5—C6	1.375 (4)	C16—H16C	0.9600
C5—H5	0.9300	C17—O7	1.420 (4)
C7—C12	1.394 (4)	C17—C18	1.475 (5)
C7—C8	1.395 (4)	C17—H17A	0.9700
C7—C14	1.505 (4)	C17—H17B	0.9700
C8—C9	1.401 (4)	C18—H18A	0.9600
C9—O2	1.362 (3)	C18—H18B	0.9600
C9—C10	1.390 (4)	C18—H18C	0.9600
C10—C11	1.376 (4)	O4—H4A	0.8200
C10—H10	0.9300	O6—H6	0.8200
C11—C12	1.373 (4)	O7—H7	0.8200
C11—H11	0.9300		
			
C6—C1—C2	119.6 (2)	O3—C13—O4	120.2 (2)
C6—C1—C13	120.3 (2)	O3—C13—C1	122.7 (2)
C2—C1—C13	120.1 (2)	O4—C13—C1	117.1 (2)
C1—C2—C3	118.8 (2)	O5—C14—O6	124.2 (3)
C1—C2—C8	120.7 (2)	O5—C14—C7	123.6 (2)
C3—C2—C8	120.4 (2)	O6—C14—C7	112.2 (2)
O1—C3—C4	124.3 (2)	O1—C15—H15A	109.5
O1—C3—C2	115.3 (2)	O1—C15—H15B	109.5
C4—C3—C2	120.4 (2)	H15A—C15—H15B	109.5
C5—C4—C3	120.5 (2)	O1—C15—H15C	109.5
C5—C4—H4	119.8	H15A—C15—H15C	109.5
C3—C4—H4	119.8	H15B—C15—H15C	109.5
C6—C5—C4	119.9 (3)	O2—C16—H16A	109.5
C6—C5—H5	120.0	O2—C16—H16B	109.5
C4—C5—H5	120.0	H16A—C16—H16B	109.5
C5—C6—C1	120.8 (2)	O2—C16—H16C	109.5
C5—C6—Br2	119.5 (2)	H16A—C16—H16C	109.5
C1—C6—Br2	119.73 (19)	H16B—C16—H16C	109.5
C12—C7—C8	119.5 (2)	O7—C17—C18	112.2 (3)
C12—C7—C14	120.3 (2)	O7—C17—H17A	109.2
C8—C7—C14	120.2 (2)	C18—C17—H17A	109.2
C7—C8—C9	118.9 (2)	O7—C17—H17B	109.2
C7—C8—C2	121.7 (2)	C18—C17—H17B	109.2
C9—C8—C2	119.3 (2)	H17A—C17—H17B	107.9
O2—C9—C10	123.5 (2)	C17—C18—H18A	109.5
O2—C9—C8	115.8 (2)	C17—C18—H18B	109.5
C10—C9—C8	120.7 (3)	H18A—C18—H18B	109.5
C11—C10—C9	119.5 (3)	C17—C18—H18C	109.5
C11—C10—H10	120.2	H18A—C18—H18C	109.5
C9—C10—H10	120.2	H18B—C18—H18C	109.5
C12—C11—C10	120.5 (3)	C3—O1—C15	117.1 (2)
C12—C11—H11	119.7	C9—O2—C16	118.3 (2)
C10—C11—H11	119.7	C13—O4—H4A	109.5
C11—C12—C7	120.7 (3)	C14—O6—H6	109.5
C11—C12—Br1	119.0 (2)	C17—O7—H7	109.5
C7—C12—Br1	120.2 (2)		
			
C6—C1—C2—C3	−0.7 (4)	C7—C8—C9—O2	−176.9 (2)
C13—C1—C2—C3	179.2 (2)	C2—C8—C9—O2	2.1 (4)
C6—C1—C2—C8	−179.3 (2)	C7—C8—C9—C10	2.6 (4)
C13—C1—C2—C8	0.6 (4)	C2—C8—C9—C10	−178.4 (2)
C1—C2—C3—O1	−178.8 (2)	O2—C9—C10—C11	177.7 (3)
C8—C2—C3—O1	−0.2 (3)	C8—C9—C10—C11	−1.8 (4)
C1—C2—C3—C4	0.4 (4)	C9—C10—C11—C12	−0.3 (5)
C8—C2—C3—C4	179.0 (2)	C10—C11—C12—C7	1.7 (4)
O1—C3—C4—C5	179.2 (3)	C10—C11—C12—Br1	179.0 (2)
C2—C3—C4—C5	0.0 (4)	C8—C7—C12—C11	−0.8 (4)
C3—C4—C5—C6	−0.1 (4)	C14—C7—C12—C11	−179.5 (3)
C4—C5—C6—C1	−0.2 (4)	C8—C7—C12—Br1	−178.13 (19)
C4—C5—C6—Br2	−178.3 (2)	C14—C7—C12—Br1	3.2 (3)
C2—C1—C6—C5	0.7 (4)	C6—C1—C13—O3	−91.2 (3)
C13—C1—C6—C5	−179.2 (3)	C2—C1—C13—O3	88.9 (3)
C2—C1—C6—Br2	178.67 (19)	C6—C1—C13—O4	89.8 (3)
C13—C1—C6—Br2	−1.2 (3)	C2—C1—C13—O4	−90.1 (3)
C12—C7—C8—C9	−1.3 (4)	C12—C7—C14—O5	70.9 (4)
C14—C7—C8—C9	177.4 (2)	C8—C7—C14—O5	−107.8 (3)
C12—C7—C8—C2	179.7 (2)	C12—C7—C14—O6	−108.4 (3)
C14—C7—C8—C2	−1.6 (4)	C8—C7—C14—O6	73.0 (3)
C1—C2—C8—C7	−100.4 (3)	C4—C3—O1—C15	−1.3 (4)
C3—C2—C8—C7	81.1 (3)	C2—C3—O1—C15	177.9 (2)
C1—C2—C8—C9	80.6 (3)	C10—C9—O2—C16	3.2 (4)
C3—C2—C8—C9	−97.9 (3)	C8—C9—O2—C16	−177.3 (3)

### Hydrogen-bond geometry (Å, °)

**Table:**

*D*—H···*A*	*D*—H	H···*A*	*D*···*A*	*D*—H···*A*
O4—H4A···O7	0.82	1.79	2.594 (3)	165
O6—H6···O3^i^	0.82	1.89	2.711 (3)	174
O7—H7···O5^ii^	0.82	2.07	2.879 (3)	167
O7—H7···Br1^ii^	0.82	3.04	3.445 (2)	113

Symmetry codes: (i) −*x*+2, −*y*+1, −*z*+1; (ii) *x*−1, *y*, *z*.
